# Dry Matter Accumulation, Water Productivity and Quality of Potato in Response to Regulated Deficit Irrigation in a Desert Oasis Region

**DOI:** 10.3390/plants13141927

**Published:** 2024-07-12

**Authors:** Hengjia Zhang, Xietian Chen, Daoxin Xue, Wanheng Zhang, Fuqiang Li, Anguo Teng, Changlong Zhang, Lian Lei, Yuchun Ba

**Affiliations:** 1 College of Agronomy and Agricultural Engineering, Liaocheng University, Liaocheng 252059, China; gsaucxt@163.com; 2 College of Water Conservancy and Hydropower Engineering, Gansu Agricultural University, Lanzhou 730070, China; tl2024202405@126.com (D.X.); 15214097901@163.com (W.Z.); lifq@gsau.edu.cn (F.L.); 3 Yimin Irrigation Experimental Station, Hongshui River Management Office, Minle, Zhangye 734500, China; tenganguo@126.com (A.T.); zcl13830689195@163.com (C.Z.); cxt19952024@126.com (L.L.); bayuchun@163.com (Y.B.)

**Keywords:** water deficit, sub-membrane drip irrigation, water consumption, water use efficiency, tuber quality, comprehensive evaluation

## Abstract

As one of the most important food crops, the potato is widely planted in the oasis agricultural region of Northwest China. To ascertain the impact of regulated deficit irrigation (RDI) on various facets including dry matter accumulation, tuber yield, quality and water use efficiency (WUE) of potato plants, a two-growth season field experiment under mulched drip irrigation was conducted in the desert oasis region of Northwest China. Water deficits, applied at the seedling, tuber formation, tuber expansion and starch accumulation stages, encompassed two distinctive levels: mild (55–65% of field capacity, F_C_) and moderate (45–55% F_C_) deficit, with full irrigation (65–75% F_C_) throughout the growing season as the control (CK). The results showed that water deficit significantly reduced (*p* < 0.05) above-ground dry matter, water consumption and tuber yield compared to CK, and the reduction increased with the increasing water deficit. A mild water deficit at the tuber formation stage, without significantly reducing (*p* > 0.05) yield, could significantly increase WUE and irrigation water use efficiency (IWUE), with two-year average increases of 25.55% and 32.33%, respectively, compared to CK. Water deficit at the tuber formation stage increased starch content, whereas water deficit at tuber expansion stage significantly reduced starch, protein and reducing sugar content. Additionally, a comprehensive evaluation showed that a mild water deficit at the tuber formation stage is the optimal RDI strategy for potato production, providing a good balance between yield, quality and WUE. The results of this study can provide theoretical support for efficient and sustainable potato production in the desert oasis regions of Northwest China.

## 1. Introduction

Agriculture is the most basic sector of material production and the foundation of the national economy, and water resources are essential to ensure agricultural production. In China, more than 60% of water consumption is used for agriculture, of which about 90% is used for irrigation [1,2,3]. In recent years, with the increase in population and water demand as well as climate change, water scarcity has become a major factor restricting agricultural production, seriously threatening the sustainable development of China’s agriculture [4,5,6]. Especially in the arid desert regions of Northwest China, irrigation is the main way to provide water consumption for crop growth [7]. However, the increasing expansion of irrigated cropland and high water-consuming crop species has further aggravated the conflicts over agricultural water use in this region [8]. Therefore, the development of efficient water-saving irrigation technologies to improve water productivity is an inevitable choice for ensuring sustainable agricultural production in the arid regions.

As one of the world’s most important food crops, potato (*Solanum tuberosum* L.) plays an irreplaceable role in ensuring food security [9]. China is the world’s largest producer of potato, with the world’s largest area under cultivation and total production [10]. The Hexi Oasis region of Northwest China has a cold climate and abundant light, which is suitable for potato growth, and is one of the main growing areas for potato [11]. Previous studies have shown that the potato plant is more sensitive to soil moisture than other food crops due to its shallow root system and smaller root mass [6,12]. Typically, the water requirement of potato plants ranges from 400 to 800 mm throughout the growing season [13,14]. Rainfall is scarce (≤300 mm) and inter-annual variability is great in the Hexi Oasis region of Northwest China, so that in almost all years the rainfall is insufficient to meet the normal growth requirements of potato plants. Therefore, irrigation becomes a necessary measure to ensure the safe production of potatoes [15]. However, the vast majority of potato growing areas in the region still use flood and furrow irrigation to deliver water to the crop. Such crude irrigation practices result in significant water wastage and scarcity, which can easily lead to reduced yields and crop failure. Improving water productivity by optimizing the irrigation mode is therefore essential for efficient potato production in arid oasis region of Northwest China.

Among several common irrigation strategies, regulated deficit irrigation (RDI) is considered as a promising water-saving technology [16,17,18]. This technique is based on the principle of applying a certain degree of water stress during crop growth, thereby regulating the nutritional and reproductive growth of the crop, and ultimately achieving the objectives of increasing water productivity and improving crop fruit quality without significantly affecting yield [19,20]. As there are differences in crop sensitivity to soil moisture at different growth stages, a clear understanding of crop response to irrigation periods and levels is crucial for successful implementation of RDI [21]. Currently, many studies have applied RDI to potato cultivation in arid areas. However, the results of these studies are not consistent due to the differences in climatic conditions and soil environments in the experimental areas. Several studies have demonstrated that deficit irrigation significantly reduces potato yield despite increasing water use efficiency [22,23,24]. However, some studies have indicated that an appropriate deficit irrigation can significantly increase water use efficiency and improve the tuber nutritional quality while maintaining potato yield [25,26]. Jensen et al. [27] showed that 30% was the limit of water saving for RDI compared to full irrigation during the whole growth phase of potato. The optimal RDI strategy to maintain potato yield was 70% of full irrigation at the end of tuber formation and 50% of full irrigation in the first two weeks at the end of the growing season. Wagg et al. [12] revealed that water deficit at any growth stage in potato negatively affected biomass, yield and tuber quality. Kifle et al. [28] indicated that potato tuber yield significantly decreased with increasing water deficit, but applying 75% of the crop water requirement during the entire growing season showed better water use efficiency. In summary, the effect of RDI on potato growth and yield formation varies considerably in different research settings. This means that RDI strategies in one region are unlikely to be transferable to other regions, and that efficient and appropriate RDI strategies need to be identified through local field trials over many years [29].

Several previous studies have reported the response mechanism of potato production to RDI; however, it has not been possible to identify the optimal RDI strategy for potato plants in the desert oasis region of Northwest China based on the current scarce research, especially under the condition of drip irrigation under a membrane. We hypothesized that excessive and insufficient irrigation is detrimental to efficient potato production in arid regions, whereas an appropriate water deficit at a certain stage of potato plant growth not only maintains yield but also improves water use efficiency (WUE) and tuber quality. Therefore, this study quantified the response of potato plant dry matter accumulation, water productivity and tuber quality to regulated deficit irrigation based on a field experiment with drip irrigation under mulch. Specifically, the precise objectives were to determine the following: (1) the characteristics of above-ground dry matter accumulation under different irrigation levels using logistic regression equations; (2) the effects of water deficit on potato water consumption (ET), yield, tuber quality and WUE at different growth stages and (3) the optimal RDI strategy based on entropy weight and technique for order preference by similarity to an ideal solution (TOPSIS) methods. The results of this study may provide useful information for researchers and farmers to implement water-saving irrigation in potato cultivation in the desert oasis region of Northwest China.

## 2. Results

### 2.1. Above-Ground Dry Matter Accumulation Characteristics

#### 2.1.1. Above-Ground Dry Matter at Different Stages

**Figure 1:**
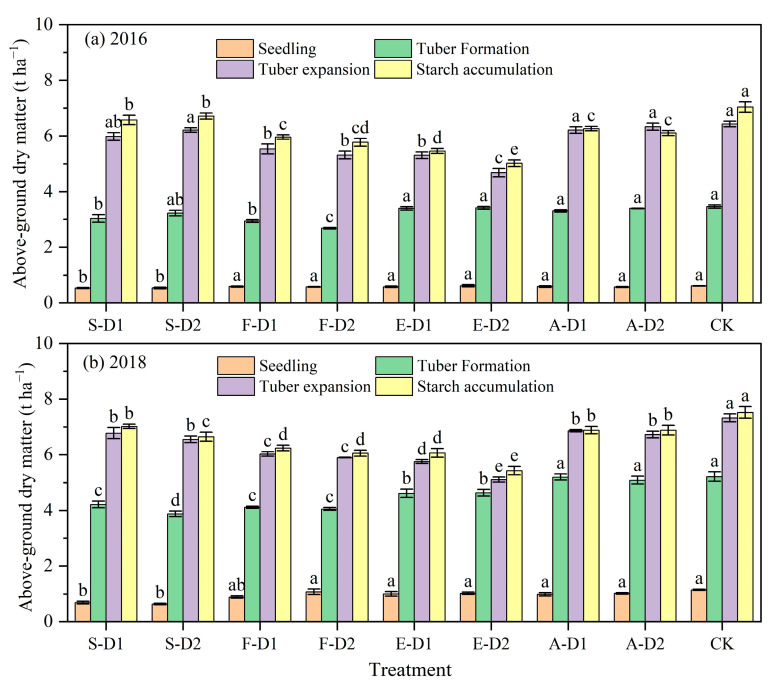
Above-ground dry matter accumulation characteristics of potato plants at different stages under each water deficit treatment in 2016 (**a**) and 2018 (**b**). Different lowercase letters indicate significant differences between treatments at the *p* < 0.05 level. The bars represent the standard deviation.

As shown in Figure 1, above-ground dry matter accumulation of potato plants under different water deficit treatments showed an increasing trend during the growing season. At the seedling stage, above-ground dry matter of the potato plant was generally low, ranging from 0.5 to 1.2 t ha^−1^. Above-ground dry matter in S-D1 (mild water deficit at the seedling stage) and S-D2 (moderate water deficit at the seedling stage) was significantly reduced (*p* < 0.05) by an average of 26.52% and 28.51%, respectively, compared to CK (full irrigation), while the rest of the water deficit treatments were not significantly different (*p* > 0.05) from CK. At the tuber formation stage, above-ground dry matter was significantly decreased by an average of 18.14% and 22.46% in F-D1 (mild water deficit at the tuber formation stage) and F-D2 (moderate water deficit at the tuber formation stage), respectively, compared to CK. At tuber expansion stage, E-D1 (mild water deficit at the tuber expansion stage) and E-D2 (moderate water deficit at the tuber expansion stage) showed a significant reduction in above-ground dry matter, averaging 19.54% and 28.77%, respectively, compared to CK. Meanwhile, above-ground dry matter in S-D1, S-D2, F-D1, and F-D2 treatments remained significantly lower than that in CK, suggesting that the effect of water deficit at the seedling and tuber formation stages on above-ground dry matter accumulation would persist even if full irrigation was resumed at later stages. At the starch accumulation stage, the above-ground dry matter was significantly lower than CK in all water deficit treatments, with decreases ranging from 4.57 to 26.74% in 2016 and 6.66 to 27.83% in 2018. In conclusion, water deficit at any stage significantly reduced above-ground dry matter accumulation in potato plants, especially at moderate water deficits.

#### 2.1.2. Above-Ground Dry Matter Accumulation Rate and Its Characteristic Parameters

The dynamic changes in above-ground dry matter accumulation in potato plants under different deficit irrigation treatments were fitted using a logistic model with days after planting as the independent variable (Table 1). It can be seen that the determination coefficients (R^2^) of the logistic model equations under all treatments were all greater than 0.99, indicating that the logistic model has a high simulation accuracy. In 2016 and 2018, the time at which the maximum rate of above-ground dry matter accumulation in potato plants occurred varied from 52 to 79 d after planting under the different water deficit treatments, the duration of rapid growth ranged from 32 to 43 d, the maximum accumulation rate ranged from 0.083 to 0.165 t ha^−1^ d^−1^, and the average accumulation rate ranged from 0.036 to 0.055 t ha^−1^ d^−1^, indicating that water deficit at different growth periods had a significant effect on the accumulation rate of above-ground dry matter. Specifically, the start time of the rapid growth period of above-ground dry matter accumulation in potato plants was earliest in the E-D2 group in both years, 11.6 d and 4.5 d earlier than CK, respectively. A-D2 (moderate water deficit at the starch accumulation stage) had the highest maximum accumulation rate, which increased by 10.34% (2016) and 28.91% (2018), respectively, compared to CK. The mean rates of potato dry matter accumulation under water deficit treatments were all lower than CK, with the greatest decrease seen in E-D2, which was 28.00% (2016) and 27.27% (2018), respectively. Furthermore, the mean accumulation rate of the moderate deficit treatments was lower than that of the mild deficit treatments over the same period, indicating that the potato plant above-ground biomass decreased more significantly with the increasing water deficit.

### 2.2. Water Consumption

**Figure 2:**
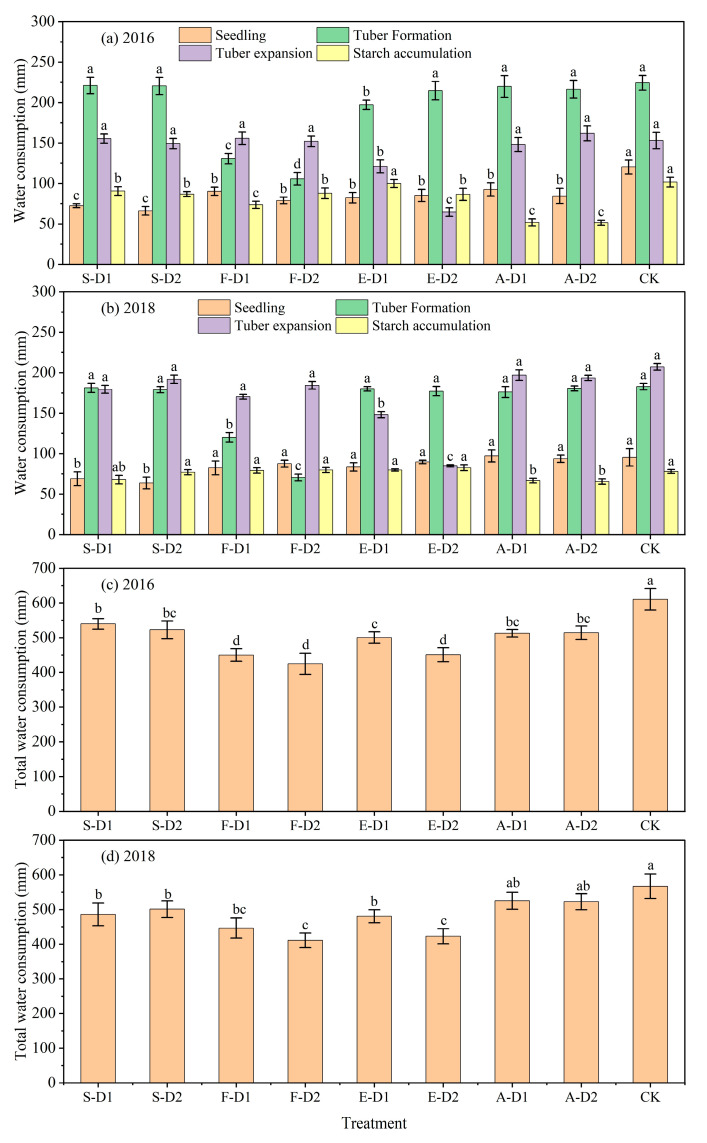
Water consumption characteristics of potato plants at different growth stages under each water deficit treatment in 2016 (**a**,**c**) and 2018 (**b**,**d**). Different lowercase letters indicate significant differences between treatments at the *p* < 0.05 level. The bars represent the standard deviation.

The ET characteristics of potato plants at different growth stages under each water deficit treatments are shown in Figure 2a,b. The ET of potato plants during the whole reproductive period showed a unimodal trend, and the peaks of ET in the two years occurred at the tuber formation and tuber expansion stages, respectively. In general, the total ET of potato plants during the whole reproductive period was 424.50–610.62 mm and 411.08–567.01 mm for all treatments in 2016 and 2018, respectively (Figure 2c,d). Compared to full irrigation, the ET of the deficit treatments was significantly reduced by 11.62–30.48% and 7.36–25.53% in the two years, respectively.

Specifically, the ET at the seedling stage was 69.62–120.26 mm (2016) and 63.66–97.16 mm (2018) for all treatments, with S-D1 and S-D2 having significantly lower (*p* < 0.05) ET than CK and other deficit treatments (Figure 2a,b). The ET at the tuber formation stage was significantly increased compared to the seedling stage, accounting for 24.90–47.61% and 17.14–41.92% of the total ET in 2016 and 2018, respectively. The ET in F-D1 and F-D2 was significantly lower than that of CK at the tuber formation stage, with a significant reduction of 38.09 and 57.17% on average in both years, respectively, while no significant difference (*p* > 0.05) was observed between the ET of the other water deficit treatments and that of CK. At the tuber expansion stage, the ET in all treatments was 64.64–161.84 mm and 84.94–207.17 mm in 2016 and 2018, respectively, accounting for approximately 14.33–35.83% and 20.09–44.88% of the total ET. The ET in E-D1 and E-D2 was significantly lower compared to other deficit treatments, with an average reduction of 24.74% and 58.38%, respectively, compared to CK. During the starch accumulation stage, the ET was significantly reduced in all treatments, accounting for 9.99–20.70% (2016) and 12.53–19.39% (2018) of the total ET. A-D1 (mild water deficit at starch accumulation stage) and A-D2 had significantly lower ET than the other treatments at the starch accumulation stage, with an average reduction of 31.77% and 32.76% compared to CK in both years, respectively. These results indicated that the degree of water deficit had a significant effect on ET at all growth stages. At the same stage, ET increased with increasing water deficit. Additionally, due to the large proportion of ET in the tuber formation and tuber expansion stages, deficit irrigation during these two periods can significantly reduce the total ET of the whole growth period.

### 2.3. Tuber Yield, Dry Matter Content and Harvest Index

Table 2 shows the effect of each water deficit treatment on potato tuber yield indexes. It can be seen that potato tuber yield varied greatly among different deficit treatments. CK had the highest yield of 36,037.23 kg ha^−1^ and 35,317.71 kg ha^−1^ in 2016 and 2018, respectively. Water deficit treatments at the seedling stage (S-D1 and S-D2) led to a reduction in tuber yield, but the difference was not significant (*p* > 0.05) compared to CK. Water deficit at the tuber formation stage (F-D1 and F-D2) significantly reduced (*p* < 0.05) potato yield compared to CK, especially by 17.58% for F-D2 treatment in 2016. The most significant effect on yield was observed for water deficit at the tuber expansion stage, which decreased by an average of 22.03% (E-D1) and 31.62% (E-D2) in 2016 and 2018, respectively, compared to CK. Potato yield was significantly lower under water deficit at the starch accumulation stage (A-D1 and A-D2) compared to CK in 2016, but the decrease was not significant in 2018. Furthermore, dry matter content of potato tubers under water deficit treatments was 20.86–22.64% (2016) and 22.25–25.84% (2018). In 2016, the dry matter content of F-D1, F-D2 and A-D1 was significantly increased by 6.59%, 4.28% and 6.40% compared with CK, respectively, and the dry matter content of other water deficit treatments was at the same level as CK. In 2018, the dry matter content of A-D2 was significantly reduced by 13.19% compared to CK, and the remaining water deficit treatments were not significantly different from CK.

As shown in Table 2, different stages and degrees of water deficit had significant effects on total potato plant biomass. Total dry biomass was highest in CK in both 2016 and 2018. Total dry biomass in S-D1 and SD2 did not show a significant difference (*p* > 0.05) compared to CK in 2016 but showed a significant decrease (*p* < 0.05) of 6.90% and 8.26% in 2018, respectively. Water deficit during the tuber formation and tuber expansion stages resulted in a significant decrease in total dry biomass, with the highest decrease in E-D2, which was significantly reduced by 39.21% (2016) and 24.11% (2018) compared to CK. In 2018, total dry biomass for water deficit during the starch accumulation stage was not significantly different from CK, while it was significantly reduced by 13.26% (A-D1) and 21.30% (A-D2) in 2016. In addition, the HI of potato plants showed significant differences between different water deficit treatments. Specifically, among all water deficit treatments, F-D1 treatment had the highest HI in both 2016 and 2018, which was significantly increased by 8.16% and 5.68% compared to CK, respectively. The difference between the HI of S-D1 and S-D2 and CK was not significant, whereas the HI of E-D1 and E-D2 was significantly reduced compared to CK, with an average decrease of 7.91% and 8.34% in both years, respectively. Water deficit during the starch accumulation stage (A-D1 and A-D2) caused a decrease in HI in both years, especially in A-D2, which was significantly reduced by 7.45% and 3.35% in 2016 and 2018 compared to CK, respectively.

### 2.4. Water Use Efficiency (WUE) and Irrigation Water Use Efficiency (IWUE)

**Figure 3:**
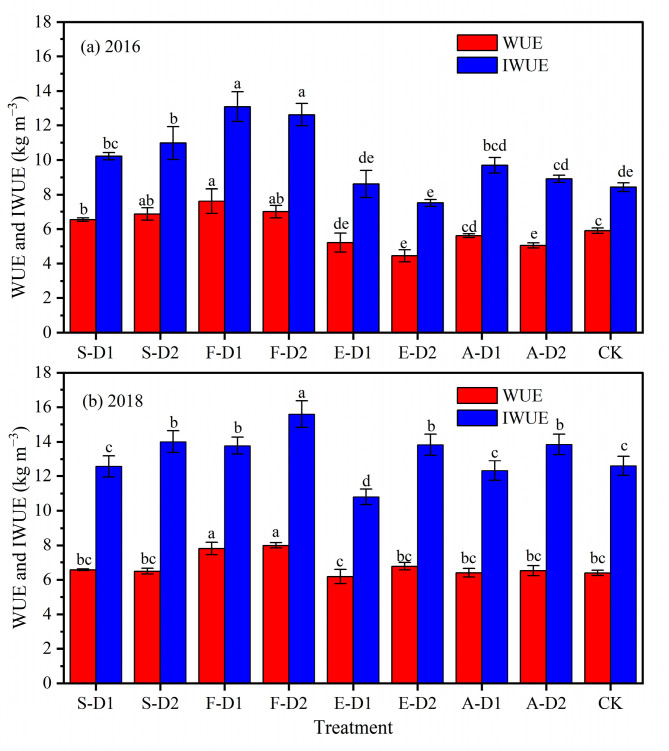
Effect of different water deficits on potato plant WUE and IWUE in 2016 (**a**) and 2018 (**b**). Different lowercase letters indicate significant differences between treatments at the *p* < 0.05 level. The bars represent the standard deviation.

Figure 3 shows the effect of different water deficits on the WUE and IWUE of potato plants. In 2016, water deficit at both the seedling and tuber formation stages significantly increased (*p* < 0.05) WUE and IWUE. Among them, WUE and IWUE were the highest in F-D1 and F-D2, with a significant increase of 29.04% and 18.59% in WUE and 55.30% and 49.75% in IWUE, respectively, compared to CK. Water deficit at the tuber expansion and starch accumulation stages did not significantly increase (*p* > 0.05) WUE and IWUE, and may even lead to a decrease, such as for the WUE and IWUE in F-D2 which were decreased by 24.60% and 10.89% compared to CK, respectively. In 2018, WUE in F-D1 and F-D2 was significantly higher than CK with 22.07% and 25.03%, respectively, while the WUE in the other water deficit treatments was at the same level as CK. Additionally, the IWUE in all moderate deficit treatments was significantly higher than CK in 2018, with F-D2 having the highest IWUE with a significant increase of 23.83% over CK. Conversely, E-D1 had the lowest IWUE, which was significantly lower than CK by 14.22%.

### 2.5. Tuber Quality

**Figure 4:**
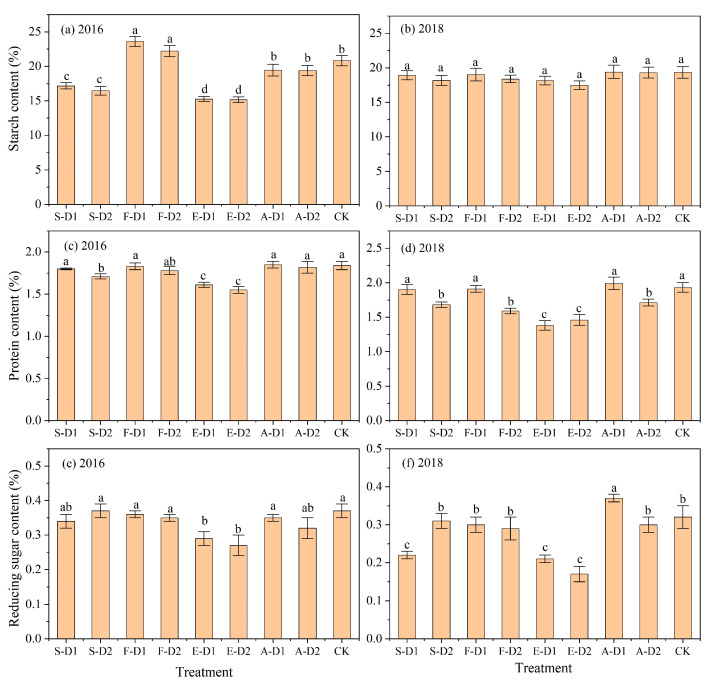
Effect of different water deficits on starch (**a**,**b**), protein (**c**,**d**) and reducing sugar (**e**,**f**) content of potato tubers in 2016 and 2018. Different lowercase letters indicate significant differences between treatments at the *p* < 0.05 level. The bars represent the standard deviation.

The effect of each water deficit treatment on potato starch content (SC), protein content (PC) and reducing sugar content (RSC) is shown in Figure 4. In 2016, SC for water deficit treatments at the tuber formation stage (F-D1 and F-D2) was significantly higher (*p* < 0.05) than CK and other treatments, while SC for water deficit treatments at the seedling (S-D1 and S-D2) and tuber expansion (E-D1 and E-D2) stages was significantly lower than CK, with decreases ranging from 17.63% to 27.63%. In 2018, there was no significant difference (*p* > 0.05) in SC between all water deficit treatments and CK. In 2016, water deficit at the tuber expansion stage resulted in a significant average reduction in PC and RSC of 14.13% and 25.64%, respectively, compared to CK, while the remaining water deficit treatments did not differ significantly from CK. In 2018, PC in S-D1, F-D1 and A-D1 was not significantly different from CK, while PC in the remaining treatments was significantly lower than CK by 12.95–28.50%. Furthermore, A-D1 exhibited the highest RSC, which was significantly higher by 6.25% compared to CK in 2018. RSC of S-D1, E-D1 and E-D2 was significantly lower compared to CK, with decreases of 31.25%, 34.38% and 46.88%, respectively.

### 2.6. Optimization of Potato Water Deficit Strategy Based on Entropy Weight and TOPSIS

Using the nine water deficit treatments undertaken in this experiment as feasible scenarios, eight indicators—yield (X1), HI (X2), WUE (X3), IWUE (X4), above-ground dry matter accumulation (X5), SC (X6), PC (X7) and RSC (X8)—were selected for the comprehensive evaluation of the multi-objectives. Firstly, the original evaluation matrix was constructed using the participating indicators and normalized. Subsequently, the weights of each of the indicators were obtained utilizing the entropy weighting method (the weights of X1–X8 were, in order, 0.129, 0.120, 0.125, 0.122, 0.129, 0.117, 0.129 and 0.13 in 2016 and 0.127, 0.115, 0.111, 0.129, 0.130, 0.128, 0.128 and 0.133 in 2018). Finally, the comprehensive score for each treatment was calculated using TOPSIS as shown in Table 3. In both 2016 and 2018, the F-D1 treatment had the highest comprehensive scores of 0.897 and 0.738, respectively. Consequently, F-D1 treatment emerges as the optimal deficit irrigation strategy that can balance multiple objectives.

## 3. Discussion

### 3.1. Effects of Water Deficit on Potato Plant Above-Ground Dry Matter, Yield and Water Use Efficiency

In arid farming areas, water is the most critical constraint to efficient crop production. Achieving higher crop yields, economic efficiency and water productivity have become the main objectives of modern agriculture [30]. Numerous studies have shown that drip irrigation techniques, especially drip irrigation under mulch, can achieve higher potato tuber yields and crop water use efficiency [31,32]. In the present study, it was found that with plastic film-covered drip irrigation, water deficit treatments caused a reduction in above-ground dry matter and its accumulation rate and tuber yield in potato plants compared to fully irrigated plants, and the reduction increased with worsening water deficit. Consistent results were also obtained from many previous studies, which revealed that water deficit had a negative effect on above-ground biomass and tuber fresh and dry yields [24,28,33,34]. The reduction in potato yield due to water deficit can be mainly attributed to its inhibition of above-ground nutrient growth. When a potato plant experiences water stress, its leaf chloroplasts are damaged and net photosynthetic rate decreases, and photosynthesis provides the basic source of material for tuber yield formation. Consequently, the decline in above-ground biomass in turn limits potato production [12,35]. Moreover, the degree of water deficit is an important factor affecting tuber yield. Martínez-Romero et al. [33] observed that a 20% reduction in water input from the normal water supply resulted in a 5% reduction in tuber yield. Greenwood et al. [36] revealed that when the water deficit exceeded 35% of the maximum allowable value, the growth rate of the potato was significantly reduced, ultimately leading to lower yields.

Improving WUE in potato plants is key to ensuring high yields, reducing production costs and minimizing negative environmental impacts [23,37]. Previous studies have shown that although water deficit leads to lower potato yields, it increases WUE due to reduced water inputs [24]. In this study, water deficit at the seedling and tuber formation stages significantly increased (*p* < 0.05) WUE and IWUE without significantly reducing yield. However, water deficit at the tuber expansion stage significantly reduced yield, resulting in a significant decrease in WUE and IWUE. This is in agreement with the findings of Li et al. [26] who found that potato plants have a high water requirement during the tuber expansion stage and water deficit at this stage had a significant effect on tuber yield, causing a significant decrease in WUE and IWUE. Furthermore, a previous study recommended that in order to improve water productivity in arid areas, water deficits should be applied during the nutritive growth and maturity stages of the potato plant and that water deficits should be avoided during the tuber expansion stage [33].

### 3.2. Effect of Water Deficit on Tuber Quality

With the continuous development of the potato processing industry, the quality of potato tubers has been allocated increasingly more attention. The main component in the dry matter of potato tubers is starch, with additional small amounts of sugar and protein [24]. Previous studies have shown that water supply has a significant effect on potato tuber quality [38]. In this study, water deficit at the tuber formation stage did not reduce potato tuber SC, and even mild water deficit at this stage can significantly increase (*p* < 0.05) SC. This result is similar to that of Carli et al. [39], who showed that potato tuber SC increased with decreasing irrigation volume. Additionally, the present study identified that water deficit at the seedling and tuber expansion stages led to a decrease in tuber SC, and the decrease with water deficit at the tuber expansion stage was particularly significant, whereas water deficit at the starch accumulation stage had no significant effect on potato tuber quality. Consistent results were obtained by Xue et al. [40] who indicated that reduced water supply at the seedling and tuber expansion stages had a significant negative effect on tuber SC.

The present study found a gradual decrease in tuber PC with increasing water deficit, with the greatest decrease seen with a moderate water deficit at the tuber expansion stage. Similarly, previous studies have found that a water deficit generally causes a decrease in plant N concentration and the decrease increases with decreasing irrigation levels [41]. Furthermore, sugar content is one of the important indicators of potato tuber quality, which is generally regulated by variety, growing environment and water management [42]. In the present study, it was found that water deficit at the tuber expansion stage resulted in a significant decrease (*p* < 0.05) in RSC, whereas the effect of water deficit at other stages was not significant (*p* > 0.05). Similar results were reported by Wegener et al. [43] who found that the total sugar, glucose and fructose contents of potato tubers under water stress were lower than those of fully irrigated tubers. Contrarily, Eldredge et al. [44] showed that water deficit increased sugar content in tubers. This divergence from the present study’s results may be attributed to the inconsistent response of different genotypes to the same treatments, that is, the response of potato tubers to water stress is not singular and uniform [45,46].

### 3.3. Comprehensive Evaluation Based on Entropy Weight and TOPSIS

With a single analysis it is difficult to identify a water management strategy that simultaneously balances yield, quality and resource use efficiency. Therefore, in the current study, a comprehensive multi-objective evaluation was performed using TOPSIS with different water deficit treatments as evaluation objects. In the process of determining the optimal water supply strategy by using TOPSIS, it is extremely critical to determine the weights of the participating indicators. In several previous studies, the weights of the evaluation indicators were set equal, which may affect the accuracy of the evaluation results [47,48]. Therefore, in order to make the evaluation results more reasonable and reliable, the entropy weight method is used in this study to objectively assign weights to the evaluation indicators. This would go some way towards removing the bias of subjective factors from the evaluation results. The results of the combined evaluation utilizing the entropy weight method and TOPSIS showed that the comprehensive score of F-D1 was optimal in both years, indicating that a mild water deficit at the tuber formation stage could better achieve the goals of stable yield, quality improvement and higher water productivity. Additionally, the comprehensive scores of E-D1 and E-D2 were in the bottom two in both years, indicating that water deficit at the tuber expansion stage would lead to a decrease in tuber yield, quality and WUE, thus suggesting that water deficit at this stage should be avoided.

## 4. Materials and Methods

### 4.1. Experimental Site Description

**Figure 5:**
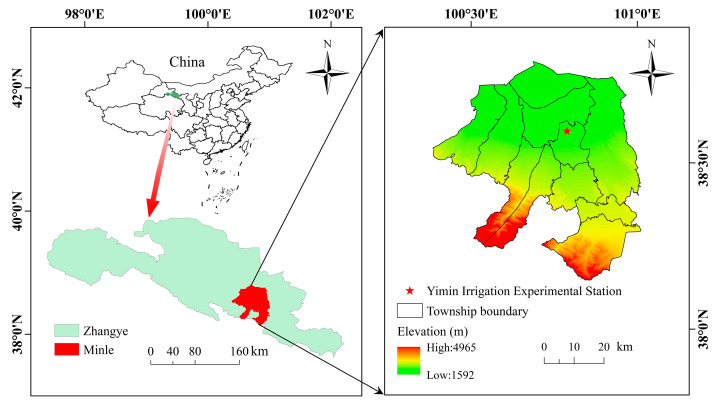
Location of the experiment station.

A field experiment, spanning two growing seasons (2016 and 2018), was executed at the Yimin Irrigation Experimental Station (100°47′ E, 38°35′ N, 1977 m a.s.l.) in Minle County, Zhangye City, Northwest China (Figure 5). The region has a cool climate and sufficient light, creating favorable conditions for photosynthesis and organic matter accumulation in crops. The multi-year average total precipitation in this site is less than 200 mm, which is far from meeting the crop’s water requirements, thus irrigation is a necessary measure to ensure normal crop production. In addition, the average annual temperature at the test site was about 6.0 °C, the frost-free period was in the range of 109–174 d and the number of sunshine hours was about 3000 h per year. The soil texture was loam, with a field capacity (F_C_) of 24.0% in the tillage layer (0–60 cm), an average soil bulk density of 1.48 t m^−3^, a pH of 8.2 and a water table greater than 20 m. The content of ammonium nitrogen, available phosphorus and available potassium in the 0–20 cm soil layer was 3.56 mg kg^−1^, 17.12 mg kg^−1^ and 81.41 mg kg^−1^, respectively.

**Figure 6:**
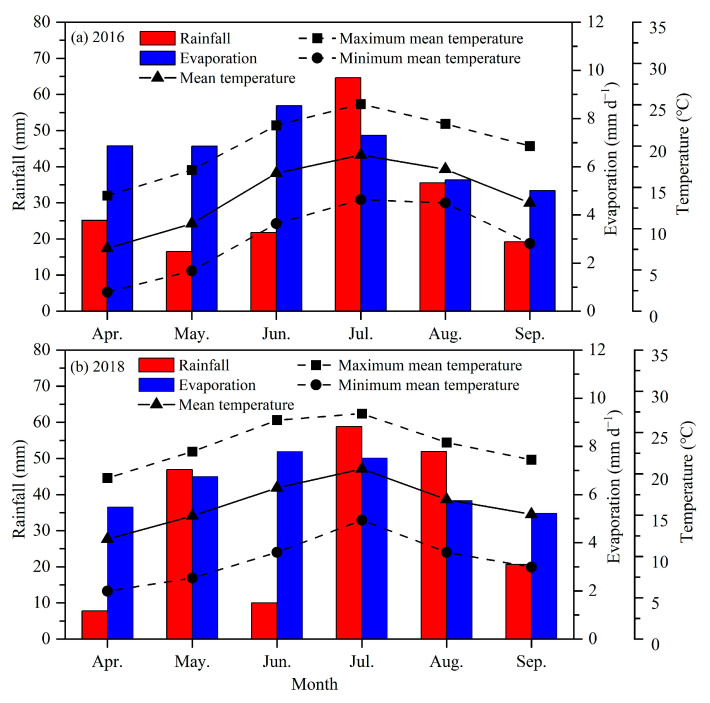
Monthly rainfall, mean evaporation and mean temperature during the potato growing season at the experimental site in 2016 (**a**) and 2018 (**b**).

Rainfall, evaporation and temperature during the two potato growing seasons are shown in Figure 6. The total rainfall during the potato growing season was 182.7 mm and 197.4 mm in 2016 and 2018, respectively. The mean daily evaporation was 6.64 mm d^−1^ in 2016 and 6.42 mm d^−1^ in 2018. The mean air temperature in 2016 was 14.02 °C, with a mean maximum of 20.23 °C and a mean minimum of 8.77 °C. The mean air temperature in 2018 was 16.32 °C, with a mean maximum temperature of 23.58 °C and a mean minimum temperature of 9.55 °C.

### 4.2. Experimental Design and Field Management

**Figure 7:**
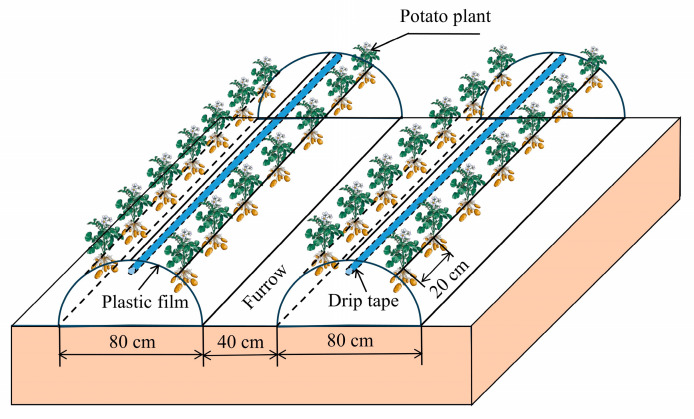
Schematic layout of drip irrigation under film for potato plants.

The potato cultivar “Qingshu168” was selected for this experiment, which has a long fertility cycle, strong adaptability, disease resistance, storage resistance and is mainly used for making starch and food processing. In 2016 and 2018, potatoes lasted 172 d and 170 d from planting (8 April 2016 and 11 April 2018) to harvesting (27 September 2016 and 28 September 2018), respectively. In this experiment, the planting mode was a combination of ridging and covering with white plastic film, in which the film width was 120 cm, the thickness was 0.01 mm, the width of the ridges was 80 cm, the height of the ridges was 20 cm, and the width of the furrow between the two ridges was 40 cm (Figure 7). Two rows of potatoes were planted on each ridge, with a spacing of 40 cm × 20 cm (plants × rows). The irrigation method used was drip irrigation with drip tape placed under the film and in the middle of the two rows of potatoes, with drippers spaced 30 cm apart and a nominal flow rate of 2.0 L h^−1^. To ensure an accurate water supply, a ball valve and water meter were installed at the water inlet of each plot. In addition, the same amount of diamine phosphate (90 kg ha^−1^), urea (120 kg ha^−1^) and potassium fertilizer (120 kg ha^−1^) were evenly spread in each plot at planting.

The entire growth period of the potato plant, dictated by its growth characteristics, was segmented into four distinct stages: seedling (S), tuber formation (F), tuber expansion (E) and starch accumulation (A). Three irrigation levels were defined in this experiment, specifically fully irrigation (65–75% F_C_), mild deficit (55–65% Fc, D1) and moderate deficit (45–55% F_C_, D2). Water deficit was applied at each growth stage, for a total of eight water deficit treatments, and full irrigation was applied during the whole growth stage as a control treatment (CK), as shown in Table 4 for the specific scheme. The experiment was performed in a single factor randomized complete block design with three replicates per treatment for a total of 27 plots. Each plot was 24 m^2^ (2.4 m × 10 m) in size, with a 1.0 m wide isolation zone between neighboring plots. For each irrigation event, the amount of water applied is based on the soil moisture content at the depth of the planned wetted layer (40 cm at seedling stage and 60 cm at other stages). Specifically, the moisture content of the soil in the planned wetted layer is measured using the dry method every approximately 7 days, and if the observed value is close to or below the designed lower limit, irrigation is immediately applied to increase the soil moisture content to the designed upper limit.

### 4.3. Measurements, Calculations and Methodologies

#### 4.3.1. Dry Matter Accumulation

Three potato plants were randomly sampled from each plot at the end of each growth stage. After cleaning the attached soil, the above-ground part (stems and leaves) and the underground part (roots and tubers) were placed in an oven at 105 °C for 30 min, followed by drying each organ to a constant weight at 75 °C. Dry matter accumulation per hectare was the average dry weight of individual plants multiplied by the planting density and the emergence rate.

In order to explore the accumulation characteristics of potato dry matter under different water deficits, the logistic model was used to nonlinearly fit the changes in above-ground dry matter during the growing period. The equation of the logistic model is as follows [49]:

(1) $$Y=\frac{K_{m}}{1+{\mathrm{ae}}^{-\mathrm{bt}}}$$

where *Y* is the observed value of potato plant above-ground biomass at a given stage (t ha^−1^); *K_m_* is the theoretical maximum value of potato plant above-ground biomass (t ha^−1^); *t* is the number of days after planting (d) and *a* and *b* are the specific fitting parameters of the equation.

The maximum accumulation rate of potato plant dry matter (*V_max_*) (t ha^−1^ d^−1^) and its corresponding time (*T_max_*) can be obtained by taking the first order derivation of the logistic equation and setting it to 0. Then, by taking the derivative of the second order and setting it to 0, the start time (*T*_1_) and the end time (*T*_2_) of the rapid growth period can be determined. The detailed equations are as follows:

(2) $$V_{\max}=\frac{K_{m}\times b}{4}$$

(3) $$T_{\max}=\frac{\ln\;a}{b}$$

(4) $$T_{1}=\frac{1}{b}\times \ln\;(\frac{a}{2+\sqrt{3}})$$

(5) $$T_{2}=\frac{1}{b}\times \ln\;(\frac{a}{2-\sqrt{3}})$$

#### 4.3.2. Tuber Yield

At harvest, fresh potatoes from an area of 2.4 m × 3 m were selected from each plot and weighed. The tuber yield was then calculated for each plot and per hectare. Harvest index (HI) was calculated by dividing the dry tuber mass by the sum of the dry tuber mass and the dry above-ground mass [50].

#### 4.3.3. Tuber Quality

After ripening, five potato tubers from each plot were sampled for quality determination. Three replicates were made for each sample. The starch content was determined using iodine colorimetry, the reducing sugar content was determined using 3,5-dinitrosalicylic acid colorimetry, and the protein content was determined using the Coomassie Brilliant Blue G-250 method [51].

#### 4.3.4. Water Consumption

The ET of potato plants at each growth stage is determined by the following water balance equation [52]:

(6) ET=P+I+K−C+ΔW

(7) $$\Delta W=10\times \sum_{i=1}^{n}\gamma_{i}H_{i}(W_{i1}-W_{i2})$$

where *P* is the effective rainfall (mm); if the individual rainfall is less than 5 mm, *P* is ignored. *I* is the irrigation amount (mm). *K* and *C* are groundwater recharge and deep seepage, respectively. Since drip irrigation was used for water supply in the test, and the groundwater depth exceeded 30 m, seepage loss and groundwater recharge could not be generated, so the values of *K* and *C* were 0. ∆*W* indicates the change in soil water storage over a period of time. *n* is the number of soil layers. In this study, the drying method was used to measure the soil water content at the depth of 0–100 cm at the interval of 20 cm soil layers, so the value of *n* was 5. *γ_i_* is the soil bulk density (g cm^−3^). *H_i_* is the thickness of the layer (20 cm). *W_i_*_1_ and *W_i_*_2_ are the soil water content (mass water content) at the beginning and end of a certain stage, respectively.

#### 4.3.5. Water Use Efficiency and Irrigation Water Use Efficiency

*WUE* and *IWUE* are calculated using the following formula [47]:

(8) $$WUE=\frac{Y}{ET}$$

(9) $$IWUE=\frac{Y}{I}$$

where *Y* is the potato tuber yield (kg ha^−1^); *ET* is crop water consumption (mm) and *I* is the total irrigation amount during the potato growing season (mm).

#### 4.3.6. Multi-Objective Optimization Based on Entropy Weight Method and TOPSIS

Entropy weighting is an objective weighting method, which determines the weight of each index according to the information provided by the observed value of each index. TOPSIS is a commonly used multi-objective comprehensive optimization method, and its decision-making basis is to compare the distance (i.e., the closeness degree) between the alternative scheme and the optimal target and the worst target. In this study, a comprehensive evaluation system was established by considering potato plant growth, yield, quality and water use efficiency indicators to obtain a multi-objective optimal deficit irrigation scheme. The specific steps are as follows [53]:

(1) Construct the original matrix.

(10) $$R={\left(r_{ij}\right)}_{m\times n}=\left[\begin{array}{ccc} \begin{array}{cc} r_{11} & r_{12}\\ r_{21} & r_{22} \end{array} & \begin{array}{c} \ldots \\ \ldots \end{array} & \begin{array}{c} r_{1n}\\ r_{2n} \end{array}\\ \begin{array}{cc} \vdots & \vdots \end{array} & \ddots & \vdots \\ \begin{array}{cc} r_{m1} & r_{m2} \end{array} & \cdots & r_{mn} \end{array}\right]$$

where *r_ij_* denotes the observed value of the evaluation indicator, and *m* and *n* are the number of treatments and indicators, respectively;

(2) Determination of weights using entropy weighting.

The dimensionless transformation of the original matrix is carried out by using the extreme value normalization method, and the matrix ***A*** is obtained. Subsequently, the entropy weight *W_j_* of each index is determined.

(11) $$A_{\mathrm{ij}}=\frac{r_{\mathrm{ij}}-\min\;(r_{i})}{\max\;(r_{i})-\min\;(r_{i})}$$

(12) $$P_{\mathrm{ij}}=\frac{A_{\mathrm{ij}}}{\sum_{i=1}^{m}A_{\mathrm{ij}}}$$

(13) $$E_{j}=-{\mathrm{Ln}(m)}^{-1}\;\sum_{i=1}^{m}P_{\mathrm{ij}}{\mathrm{Ln}(P}_{\mathrm{ij}})$$

(14) $$W_{j}=\frac{1-E_{j}}{\sum_{j=1}^{n}E_{j}}$$

where *P_ij_* is the characteristic proportion of the *i*th evaluation object on the *j*th index; *E_j_* is the entropy of the *j*th index;

(3) Multi-objective optimization using TOPSIS.

The original matrix ***R*** was normalized to obtain matrix ***B*** = [*b_ij_*]_m×n_, and then the weighted matrix ***Z*** was obtained by multiplying the entropy weight.

(15) $$b_{\mathrm{ij}}=\frac{r_{\mathrm{ij}}}{\sqrt{\sum_{i=1}^{m}r_{\mathrm{ij}}^{2}}}$$

(16) $$Z_{\mathrm{ij}}=W_{j}\times b_{\mathrm{ij}}$$

Calculation of the Euclidean distance D^+^ and D^−^ and the relative proximity (*C_i_*) between each evaluation object and the ideal solution is then performed.

(17) $$D_{i}^{+}=\sqrt{\sum_{j=1}^{n}(z_{j}^{+}-z_{\mathrm{ij}}{)}^{2}}$$

(18) $$D_{i}^{-}=\sqrt{\sum_{j=1}^{n}(z_{\mathrm{ij}}-z_{j}^{-}{)}^{2}}$$

(19) $$C_{i}=\frac{D_{i}^{-}}{D_{i}^{+}+D_{i}^{-}}$$

where $z_{j}^{+}$ is defined as a positive ideal solution, $z^{+}=\left[max(z_{1}),max(z_{2}),\cdots ,max(z_{n})\right]$, $z_{j}^{-}$ is defined as a negative ideal solution and $z^{-}=\left[min(z_{1}),min(z_{2}),\cdots ,min(z_{n})\right]$. *C_i_* is the decision basis for ranking the schemes. A value of C_i_ closer to 1 indicates a better comprehensive evaluation result.

### 4.4. Statistical Analysis

Microsoft Excel 2010 was utilized for data processing and computation. One-way analysis of variance (ANOVA) was performed using SPSS 23.0 software (SPSS Inc., Chicago, IL, USA). Mean differences were examined via the Duncan’s multiple range tests, adopting a significance level of *p* < 0.05. Origin 2021 software (OriginLab Inc., Northampton, MA, USA) was used to generate the figures.

## 5. Conclusions

Water deficit decreased above-ground biomass and its accumulation rate, tuber yield and water consumption in potato plants, and the decrease was more significant with increasing water deficit. Among all deficit treatments, water deficit at the tuber expansion stage had the greatest negative effect on potato growth, resulting in the most significant reductions (*p* < 0.05) in yield and harvest index. Water deficit at the seedling and tuber formation stages can significantly increase water use efficiency and irrigation water use efficiency while slightly reducing tuber yield. Moreover, water deficit at the tuber formation stage was beneficial for increasing starch content but had no significant effect (*p* > 0.05) on protein and reducing sugar content. On the contrary, water deficit at the tuber expansion stage significantly reduced the content of starch, protein and reducing sugar in the potato. The evaluation results using entropy weighting and TOPSIS methods showed that a mild water deficit at the tuber formation stage ranked first in the overall score, which effectively balanced multiple objectives such as yield, quality and water use efficiency. Therefore, mild water deficit at the tuber formation stage can be recommended as the optimal RDI strategy for potato cultivation in the Hexi desert oasis of northwest China. Nonetheless, the optimal RDI strategy identified in this study may not be applicable to other arid agricultural regions due to differences in soil properties and climatic conditions. Consequently, future research should be conducted in multiple regions to elucidate how different climatic variations and soil textures affect the development of optimal RDI strategies.

## Figures and Tables

**Table 1:** Parameters and eigenvalues of the logistic model for above-ground dry matter accumulation in potato plants under different water deficit treatments.

Year	Treatment	Regression Equation	R^2^	T_1_	T_2_	T_max_	∆T	V_max_	V_mean_
2016	S-D1	y = 6.57/(1 + 202.270e^−0.067x^)	0.997	59.59	98.90	79.25	39.31	0.110	0.047
	S-D2	y = 6.72/(1 + 211.388e^−0.068x^)	0.998	59.36	98.10	78.73	38.73	0.114	0.048
	F-D1	y = 5.96/(1 + 168.382e^−0.066x^)	0.997	57.72	97.62	77.67	39.91	0.098	0.042
	F-D2	y = 5.77/(1 + 195.330e^−0.067x^)	0.996	59.07	98.38	78.73	39.31	0.097	0.041
	E-D1	y = 5.45/(1 + 145.365e^−0.071x^)	0.999	51.58	88.68	70.13	37.10	0.097	0.039
	E-D2	y = 5.01/(1 + 78.941e^−0.066x^)	0.999	46.24	86.15	66.19	39.91	0.083	0.036
	A-D1	y = 6.27/(1 + 470.230e^−0.081x^)	0.995	59.71	92.22	75.97	32.52	0.127	0.044
	A-D2	y = 6.33/(1 + 428.301e^−0.081x^)	0.993	58.55	91.07	74.81	32.52	0.128	0.045
	CK	y = 7.04/(1 + 169.513e^−0.066x^)	0.999	57.82	97.73	77.77	39.91	0.116	0.050
2018	S-D1	y = 7.01/(1 + 113.236e^−0.070x^)	0.999	48.75	86.38	67.56	37.63	0.123	0.052
	S-D2	y = 6.64/(1 + 139.822e^−0.073x^)	0.999	49.64	85.72	67.68	36.08	0.121	0.049
	F-D1	y = 6.24/(1 + 62.181e^−0.066x^)	0.998	42.62	82.53	62.58	39.91	0.103	0.046
	F-D2	y = 6.05/(1 + 43.133e^−0.062x^)	0.999	39.47	81.96	60.71	42.48	0.094	0.044
	E-D1	y = 6.06/(1 + 63.009e^−0.072x^)	0.998	39.25	75.84	57.55	36.58	0.109	0.045
	E-D2	y = 5.42/(1 + 82.067e^−0.084x^)	0.997	36.79	68.15	52.47	31.36	0.114	0.040
	A-D1	y = 6.98/(1 + 88.792e^−0.076x^)	0.999	41.70	76.36	59.03	34.66	0.133	0.051
	A-D2	y = 6.88/(1 + 77.639e^−0.074x^)	0.999	41.02	76.61	58.81	35.59	0.165	0.051
	CK	y = 7.51/(1 + 62.0229e^−0.068x^)	0.999	41.33	80.07	60.70	38.73	0.128	0.055

R^2^, determination coefficient; T_1_, the start time of the rapid growth period, d; T_2_, the termination time of the rapid growth period, d; T_max_, the time corresponding to the maximum accumulation rate, d; ∆T, duration of the rapid growth period, d; V_max_, maximum accumulation rate, t ha^−1^ d^−1^; V_mean_, mean accumulation rate, t ha^−1^ d^−1^.

**Table 2:** Effect of water deficit on potato yield, tuber dry matter content, total dry biomass and harvest index. Different lowercase letters indicate significant differences between treatments at the *p* < 0.05 level.

Year	Treatment	Fresh Tuber Yield (kg ha^−1^)	Tuber Dry Matter Content (%)	Total Dry Biomass (kg ha^−1^)	Harvest Index
2016	S-D1	35,271.74 ab	21.37 b	13,312.03 a	0.566 b
	S-D2	35,834.08 a	21.15 b	13,293.98 a	0.570 b
	F-D1	34,276.71 b	22.64 a	12,726.34 b	0.610 a
	F-D2	29,701.00 c	22.15 ab	11,357.03 c	0.579 b
	E-D1	25,839.44 d	20.80 b	10,430.18 d	0.515 c
	E-D2	20,066.11 e	21.02 b	8255.62 e	0.511 c
	A-D1	28,784.62 c	22.60 a	11,780.24 c	0.552 b
	A-D2	25,975.15 d	21.47 b	10,688.34 d	0.522 c
	CK	36,037.23 a	21.24 b	13,581.47 a	0.564 b
2018	S-D1	31,913.54 ab	25.84 a	12,257.50 b	0.673 bc
	S-D2	32,508.33 ab	25.67 a	12,083.72 b	0.691 b
	F-D1	34,852.08 a	24.66 ab	11,841.06 b	0.726 a
	F-D2	32,879.17 ab	23.98 ab	11,733.67 bc	0.672 bc
	E-D1	29,747.92 b	24.01 ab	11,197.44 c	0.638 d
	E-D2	28,633.33 b	22.25 b	9996.05 d	0.637 d
	A-D1	33,619.79 a	25.19 a	13,145.88 a	0.644 d
	A-D2	34,050.00 a	24.89 ab	12,755.40 a	0.664 c
	CK	35,317.71 a	25.63 a	13,171.44 a	0.687 b

**Table 3:** Comprehensive score and ranking of different water deficit treatments based on entropy weight and TOPSIS.

Treatment	2016				2018			
	D^+^	D^−^	C_i_	Ranking	D^+^	D^−^	C_i_	Ranking
S-D1	0.021	0.051	0.711	4	0.025	0.020	0.446	7
S-D2	0.019	0.055	0.749	3	0.015	0.027	0.641	6
F-D1	0.007	0.060	0.897	1	0.011	0.031	0.738	1
F-D2	0.014	0.054	0.794	2	0.015	0.029	0.654	4
E-D1	0.037	0.039	0.514	8	0.035	0.008	0.184	9
E-D2	0.048	0.030	0.382	9	0.037	0.010	0.222	8
A-D1	0.025	0.047	0.654	6	0.015	0.034	0.695	3
A-D2	0.032	0.041	0.564	7	0.015	0.027	0.644	5
CK	0.023	0.056	0.710	5	0.014	0.033	0.699	2

D^+^/D**^−^**, Euclidean distance between the evaluation object and the positive/negative ideal value. C_i_, the relative closeness of each evaluation object.

**Table 4:** Potato deficit irrigation design scheme.

Treatment	Relative Soil Water Content (% of the Field Capacity)			
	Seedling	Tuber Formation	Tuber Expansion	Starch Accumulation
S-D1	55–65%	65–75%	65–75%	65–75%
S-D2	45–55%	65–75%	65–75%	65–75%
F-D1	65–75%	55–65%	65–75%	65–75%
F-D2	65–75%	45–55%	65–75%	65–75%
E-D1	65–75%	65–75%	55–65%	65–75%
E-D2	65–75%	65–75%	45–55%	65–75%
A-D1	65–75%	65–75%	65–75%	55–65%
A-D2	65–75%	65–75%	65–75%	45–55%
CK	65–75%	65–75%	65–75%	65–75%

S-D1, mild water deficit at seedling stage; S-D2, moderate water deficit at seedling stage; F-D1, mild water deficit at tuber formation stage; F-D2, moderate water deficit at tuber formation stage; E-D1, mild water deficit at tuber expansion stage; E-D2, moderate water deficit at tuber expansion stage; A-D1, mild water deficit at starch accumulation stage; A-D2, moderate water deficit at starch accumulation stage; CK, full irrigation throughout the growing season.

## Data Availability

The datasets used and/or analyzed during the current study are available from the corresponding author upon reasonable request and the approval of the data owner.
